# Myeloid-Derived Suppressor Cells Dampen Airway Inflammation Through Prostaglandin E2 Receptor 4

**DOI:** 10.3389/fimmu.2021.695933

**Published:** 2021-07-12

**Authors:** Chiel van Geffen, Astrid Deißler, Sandra Beer-Hammer, Bernd Nürnberg, Rupert Handgretinger, Harald Renz, Dominik Hartl, Saeed Kolahian

**Affiliations:** ^1^ Department of Experimental and Clinical Pharmacology and Pharmacogenomics, University Hospital Tübingen, Tübingen, Germany; ^2^ Institute of Laboratory Medicine and Pathobiochemistry, Molecular Diagnostics, Philipps University of Marburg, Marburg, Germany; ^3^ Universities of Giessen and Marburg Lung Center, German Center for Lung Research (DZL), Marburg, Germany; ^4^ Department of Pharmacology, Experimental Therapy & Toxicology and Interfaculty Center of Pharmacogenomics & Drug Research (IZePhA), University Hospitals and Clinics, Eberhard Karls University Tübingen, Tübingen, Germany; ^5^ Children’s University Hospital, Eberhard Karls University of Tübingen, Tübingen, Germany; ^6^ Department of Pediatrics I, Eberhard Karls University of Tübingen, Tübingen, Germany; ^7^ Translational Medicine, Novartis Institutes for BioMedical Research, Basel, Switzerland

**Keywords:** myeloid-derived suppressor cells, prostaglandin E2, E-prostanoid receptors, arginase-1, airway inflammation, asthma

## Abstract

Emerging evidence suggests a mechanistic role for myeloid-derived suppressor cells (MDSCs) in lung diseases like asthma. Previously, we showed that adoptive transfer of MDSCs dampens lung inflammation in murine models of asthma through cyclooxygenase-2 and arginase-1 pathways. Here, we further dissected this mechanism by studying the role and therapeutic relevance of the downstream mediator prostaglandin E2 receptor 4 (EP4) in a murine model of asthma. We adoptively transferred MDSCs generated using an EP4 agonist in a murine model of asthma and studied the consequences on airway inflammation. Furthermore, pegylated human arginase-1 was used to model MDSC effector activities. We demonstrate that the selective EP4 agonist L-902,688 increased the number and suppressive activity of MDSCs through arginase-1 and nitric oxide synthase-2. These results showed that adoptive transfer of EP4-primed MDSCs, EP4 agonism alone or arginase-1 administration ameliorated lung inflammatory responses and histopathological changes in asthmatic mice. Collectively, our results provide evidence that MDSCs dampen airway inflammation in murine asthma through a mechanism involving EP4.

## Introduction

Asthma is the most common chronic lung disease with an estimated prevalence of over 300 million people worldwide (1). Currently, the most common forms of anti-inflammatory treatment of asthma are corticosteroids, which are non-specific and are associated with side effects (2). Furthermore, the use of biologic therapies, such as anti-immunoglobulin (Ig) E, anti-interleukin (IL)-5 and anti-IL-4R, are limited to severe phenotypes of asthma with high eosinophil and IgE levels and restricted by the age of patients (3). The discovery of novel treatments are therefore of paramount importance.

There is an increasing amount of evidence supporting the involvement of myeloid-derived suppressor cells (MDSCs) in lung diseases like asthma (4). MDSCs represent a phenotypically heterogeneous group of immature myeloid cells that potently suppress the immune response. MDSCs were first identified in cancer patients, but their role expanded into a wide variety of inflammatory, infective and autoimmune diseases (5). MDSCs are mainly subdivided into polymorphonuclear (PMN-) and monocytic (M-) MDSCs, and in mice are characterized by CD11b^+^Ly6G^+^Ly6C^int^ and CD11b^+^Ly6G^-^Ly6C^hi^ markers, respectively (6). The expansion and activation of MDSCs is mediated by a multitude of factors, such as vascular endothelial growth factor, granulocyte macrophage colony-stimulating factor (GM-CSF), IL-4, IL-6, IL-13, interferon-gamma, cyclooxygenase-2 (COX2) and transforming growth factor β (5). MDSC-mediated immune suppression involves multiple mediators and mechanisms, with reactive oxygen species (ROS), arginase-1 and inducible nitric oxide synthases (iNOS) as the most established ones (5).

Previously, we showed that MDSCs play an important role in a murine model of asthma (7). In particular, adoptively transferred MDSCs were recruited to the lungs of asthmatic mice and reduced airway inflammatory features (7). However, the signals that drive the expansion and activation of MDSCs in lung inflammatory conditions remained incompletely defined. Unravelling these signals is crucial as MDSC-mediated immune suppression presents a potential mechanism to suppress the underlying chronic inflammation characterizing asthma. COX2 and its downstream mediator prostaglandin (PG) E2 play an important role in both the expansion and activation of MDSCs in cancer (8). PGE2 is a key immunomodulator associated with anti-inflammatory and tissue repair effects in the lung (9–12). It acts on a group of four G-protein-coupled receptors, termed E-prostanoid (EP) receptors (EP1-4) (13). Each of these EP receptors differ in intracellular signaling and response (13).

Collectively, the present study aimed to dissect the role of PGE2 and EP receptors on MDSC generation and activation in a murine model of asthma.

## Material and Methods

### Mice

Six- to eight-week-old female BALB/c mice were purchased from Charles River (Sulzfeld, Germany). All animals were housed in individually ventilated cages and provided with food and water *ad libitum* in a controlled environment. A 12 h light–dark cycle was maintained for all animals. All animal studies were reviewed and approved by the Regierungspräsidium Tübingen, Tübingen, Germany (animal protocols PH 01/19M and PH 07/19G) and were carried out according to the guidelines of the German law of protection of animal life.

### MDSC Culture

Bone marrow was collected from the hind limbs of mice (femur and tibia) and single cell suspensions were prepared followed by red blood cell lysis [155 mM ammonium chloride (Merck, Darmstadt, Germany), 10 mM potassium bicarbonate (Carl Roth, Karlsruhe, Germany) and EDTA (0.1 mM) in ddH2O]. Cells were then cultured in RPMI 1640 supplemented with 10% FBS, 2 mM L-glutamine, 1% penicillin/streptomycin, 50 µM 2-mercaptoethanol (all from Gibco, Thermo Fisher Scientific, Waltham, MA, USA) together with 20 ng/ml GM-CSF and 20 ng/ml IL-6 (BioLegend, San Diego, USA) at 37°C, 5% CO2 v/v. The following compounds were tested: PGE2, EP2 receptor agonist Butaprost, EP1/EP3 receptor agonist Sulprostone and EP4 receptor agonist L-902,688 (all from Cayman Chemical, Ann Arbor, USA). Cells were harvested after three days and subtyped for MDSCs by flow cytometry (BD FACSCanto II, BD Biosciences, Franklin Lakes, NJ, USA). Both PMN-MDSCs (based on positive selection of the Ly6G marker) and M-MDSCs (based on positive selection of the Gr1 marker of the Ly6G depleted cell suspension) were isolated from the cell suspension using the magnetic-activated cell sorting (MACS) MDSC isolation kit (Miltenyi Biotec, Bergisch Gladbach, Germany), according to manufacturer’s instruction.

### Suppression Assay

At the day of MDSC isolation, single-cell suspensions were prepared from the spleen of naïve donor mice followed by red blood cell lysis. CD4^+^ T cells were isolated using the MACS CD4^+^ T cell isolation kit (Miltenyi Biotec) and stained with 1 µmol/L 5 (6)-Carboxyfluorescein diacetate succinimidyl ester (CFSE; BioLegend), according to manufacturer’s instruction. CFSE-labeled purified CD4^+^ T cells were seeded in U-shaped 96-well plates at 1 x 10^5^ cells per well and co-cultured with isolated PMN- or M-MDSCs at different ratios (MDSC: CD4^+^ T cell at 1:1, 1:2, 1:4 and 1:8) for three days. Co-culture medium consisted of RPMI 1640 supplemented with 10% FBS, 2 mM L-glutamine, 1% penicillin/streptomycin, 50 µM 2-mercaptoethanol together with 100 U/ml IL-2 (BioLegend) and anti-biotin MACSiBead particles loaded with CD3ϵ- and CD28-biotin (Miltenyi Biotec) with a bead-to-cell ratio of 1:1. After three days of co-culture, the proliferation of CFSE-labeled CD4^+^ T cells was analyzed by flow cytometry. The relative proliferation was calculated as the percentage of proliferation relative to control (i.e., CD4^+^ T cells cultured in the absence of MDSCs, 100%).

### Murine Model of Asthma

The mouse model of allergic airway inflammation was performed as reported previously using house dust mite (HDM) (14). Briefly, HDM extracts (Greer Laboratories, Lenoir, USA) or PBS was administered intranasal on days 0 (100 µg in 20 µl PBS), 7, 8, 9, 10 and 11 (10 µg in 20 µl PBS), while control mice received the same volumes of PBS only (**Figure E4A**). Intranasal procedures were carried out under light anesthesia using isoflurane.

### MDSC Adoptive Transfer

Bone marrow cells from healthy donor mice were cultured in the presence of EP4 agonist L-902,688 (10 µM) or vehicle and after three days, both PMN- and M-MDSCs were isolated as described above. Adoptive transfer was performed on day 7 after the first HDM sensitization by intravenously transferring 2x10^6^ of either PMN- or M-MDSCs, dissolved in PBS with an end volume of 100 µl, into asthmatic mice *via* the lateral tail vein. Control mice received 100 µl PBS only. Three days after the last HDM exposure, mice were sacrificed and bronchoalveolar fluid (BALF), lung, blood, bone marrow and spleen were collected (**Figure E4A**).

### L-902,688/BCT-100 Administration

On day 7, 9 and 11, asthmatic mice were intravenously administered EP4 agonist L-902,688 (0.1; 0.2 or 0.4 mg/kg) or BCT-100 (20 mg/kg) or PBS. BCT‐100 was kindly provided by Bio‐Cancer Treatment International Limited (Hong Kong, China). Three days after the last HDM challenge, mice were sacrificed and BALF, lung, blood, bone marrow and spleen were collected (**Figure 5A**).

### Single Cell Preparation for Flow Cytometry

From each mouse, single cell suspensions of bone marrow, spleen, blood and lung were prepared. The bone marrow and spleen were processed as described previously. Blood was collected by cardiac puncture, mixed with EDTA to prevent clotting, and centrifuged (1000 g for 10 min.) to obtain the serum. Serum pellets were lysed for red blood cells to obtain peripheral blood mononuclear cells (PBMCs). The right lobe of the lung was digested using 200 U/ml Collagenase type IV, 25 U/ml DNAse I and 2.5 mM MgCl2 dissolved in Hanks’ Balanced Salt Solution (HBSS) (all from Thermo Fisher Scientific) at 37°C for 45 minutes with continuous agitation. The digested tissue was mashed through a 70 µm cell strainer twice and the red blood cells were lysed to obtain the single cell suspension from the lung.

### Flow Cytometry

Obtained single cell suspensions were analyzed by flow cytometry using the BD FACSCanto II (BD Biosciences) after staining the cells with the corresponding fluorescently labeled antibodies. MDSCs were characterized using anti-CD11b-APC (M1/70), anti-Ly6G-FITC (1A8) and anti-Ly6C-PE/Cy7 (HK1.4) (all from BioLegend) together with 7-AAD (BD Biosciences). Additionally, anti-CD84 (mCD84.7) and anti-Jaml (4E10) (both from BioLegend) were used to test for their expression in MDSCs (15). T cells were characterized using anti-CD4-APC (53-6.7), anti-CD25-FITC (3C7) and anti-CD69-PE (H1.2F3) (all from BioLegend). Surface staining was carried out in PBS supplemented with 1% BSA and 0.02% NaN_3_ (Thermo Fisher Scientific). Flow cytometry data was analyzed with FlowJo software (BD Biosciences; FlowJo LLC, Ashland, OR, USA). The gates for samples without live/dead staining were set according to their specific forward scatter and side scatter properties, to select viable cells. Independent 7-AAD staining was used to confirm the correct use of viable cell gates. For multiple fluorochrome panels, the correct compensation was applied and gates were set with the use of appropriate isotype control antibodies and fluorescence minus one controls.

### qPCR

RNA was isolated from MDSCs using the RNeasy kit (Qiagen, Venlo, Netherlands), reverse-transcribed into cDNA using the High-Capacity cDNA Reverse Transcription Kit (Applied Biosystems, Foster City, USA), and analyzed by qPCR (LightCycler 480, Roche, Basel, Switzerland) according to manufacturer’s instruction (**Table S1**).

### Histopathology

The left diaphragmatic lobe of the lungs was fixated with 4% neutral phosphate-buffered formalin. Lung sections were stained with haematoxylin and eosin (H&E) to assess perivascular and peribronchial inflammation and with periodic acid-Schiff (PAS) stain (Carl Roth, Karlsruhe, Germany) to quantify mucus-containing goblet cells. The severity of perivascular and peribronchial inflammation was evaluated by scoring the amount of inflammatory cells formed around the vessels and airways from grade 0 to grade 4 as follows: 0, normal lung; 1, scattered infiltration of a few inflammatory cells; 2, one ring of inflammatory cells; 3, two- to four layers of inflammatory cells; 4, more than four layers of inflammatory cells (16). PAS-positive mucus-containing cells were semi-quantified by scoring from grade 0 to grade 4 as follows: 0, <5% PAS-positive cells; 1, 5-25%; 2, 25-50%; 3, 50-75%; 4, >75% (16).

### BALF

For BALF collection, the tracheas were cannulated with a 20-gauge catheter, after which the lungs were lavaged three times with 0.8 ml of PBS. Supernatants were stored and the pellets prepared for Cytospin. Differential cell counts were performed on Cytospin preparations stained according to Pappenheim, with Giemsa (Merck) and May-Grünwald (Merck) stains. A total of 400 cells were counted under a light microscope to classify eosinophils.

### ELISA

Cytokines in BALF supernatants and HDM-specific IgE levels in the serum were examined using ELISAs according to manufacturer’s protocol (R&D System, Minneapolis, USA; Chondrex, Redmond, USA).

### Western Blot

Proteins from isolated PMN- and M-MDSCs were extracted. In short, cells were lysed with NP-40 lysis buffer (150 mM NaCl, 1.0% NP-40, 50 mM Trist-HCl, protease inhibitors (Carl Roth)) for 30 minutes during continuous agitation and spun down at 16000 g for 20 minutes, after which the supernatant was collected. The cell lysates were separated by 10% SDS-PAGE gels in Laemmli loading buffer, and transferred to polyvinylidene fluoride (PVDF) membranes (Invitrogen, Carlsbad, CA, USA). After blocking with 5% skim milk/PBS/0.1% Tween (Carl Roth), blots were probed with primary antibodies for the EP1-4 receptors (Alomone Labs, Jerusalem, Israel, 1:250) and GAPDH (Abcam, Cambridge, UK, 1:2500). Membranes were then incubated with the appropriate secondary horseradish peroxidase-conjugated goat anti-rabbit (Abcam, 1:1000) for 1 hour at room temperature.

### Statistics

All statistical analyses were performed in GraphPad Prism version 8.0 (San Diego, USA). Data are presented as mean ± SD unless otherwise specified. A *p* value of less than 0.05 was considered statistically significant.

## Results

### PGE2 and EP4 Agonism Enhance the Generation of MDSC

**Figure 1:**
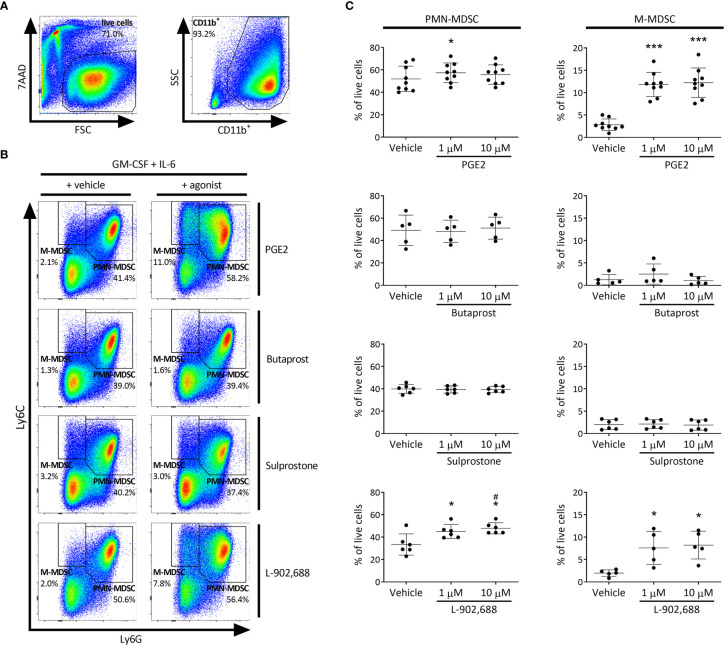
The effect of PGE2 and EP receptor agonists on MDSC generation in murine bone marrow cells. Bone marrow cells isolated from the hind legs of 6- to 8-week-old female BALB/c mice were cultured in the presence of PGE2 or EP receptor agonists, including EP2 agonist Butaprost, dual EP1/EP3 agonist Sulprostone and EP4 agonist L-902,688 (1 or 10 µM) or their respective vehicles in combination with GM-CSF (20 ng/ml) and IL-6 (20 ng/ml) for 3 days. **(A)** Gating strategy of MDSC subtyping by flow cytometry. CD11b^+^ cells were first selected from live bone marrow cells before MDSCs were gated. **(B)** Representative MDSC gating of bone marrow cells cultured in either vehicle (left) or 10 µM agonist (right), where CD11b^+^Ly6G^+^Ly6C^int^ and CD11b^+^Ly6G^-^Ly6C^hi^ are defined as PMN- and M-MDSCs, respectively. **(C)** Quantitative analysis of both PMN- (left) and M-MDSCs (right) were performed. Data shown as the percentage of live cells. Data was obtained from 5-9 independent experiments, with bone marrow cells from each mouse divided into the different conditions (vehicle, 1 or 10 µM) of each experiment, including 2 technical replicates per condition. Results of individual experiments and mean ± SD are shown. Statistical analysis was performed with repeated measures one-way ANOVA followed by Tukey’s multiple comparisons test. *p < 0.05, ***p < 0.001 compared with vehicle, ^#^ *p* < 0.05 compared to 1 µM.

To test whether PGE2 and/or selective EP receptor agonists are able to generate MDSCs, we used an adaptation of a method commonly used to induce MDSCs from bone marrow cells *in vitro* (17). By additionally exposing these bone marrow cells to PGE2 or selective EP receptor agonists during the generation process and subsequently subtyping PMN- and M-MDSC populations, we assessed their capacity to generate MDSCs. EP receptor (EP1-4) expression by MDSCs was confirmed (data not shown). The CD11b^+^Ly6G^+^Ly6C^int^ (PMN-MDSC) population significantly increased with the addition of PGE2 (at 1 µM) and the EP4 agonist L-902,688 compared to vehicle (**Figures 1A–C**). Furthermore, the addition of PGE2 and EP4 agonist L-902,688 in the culture significantly increased the CD11b^+^Ly6G^-^Ly6C^int^ (M-MDSC) population compared to vehicle. The EP2 agonist Butaprost, and the EP1/EP3 agonist Sulprostone, did not alter the generation of MDSCs.

### PGE2 Augments the Immunosuppressive Activity of MDSCs Through the EP2- and EP4 Receptor

Both PMN- and M-MDSCs were isolated from bone marrow cultures, with purities of 97.3 ± 0.8% and 81.2 ± 4.3%, respectively (**Figure E1**). As MDSCs are defined by their suppressive properties (5), we then assessed the immunosuppressive activity using a MDSC - CD4^+^ T cell co-culture suppression assay. The agonists used in the bone marrow cell culture were also added during the co-culture of MDSCs with CD4^+^ T cells in an attempt to maintain MDSC activation. The addition of PGE2, the EP2 agonist Butaprost and the EP4 agonist L-902,688 significantly decreased CD4^+^ T-cell proliferation compared to vehicle (**Figures E2A–C**).

### PGE2 Directly Inhibits T-Cell Proliferation Through EP2 and EP4 Receptor Agonism

To study the potential direct immunosuppressive effect of the PGE2 agonists, CD4^+^ T-cell suppression assays were performed in the presence of only PGE2 or EP receptor agonists. Addition of PGE2, EP2 agonist Butaprost or EP4 agonist L-902,688 alone significantly decreased CD4^+^ T-cell proliferation, while the EP1/EP3 agonist Sulprostone, did not show any direct T-cell suppressive effect (**Figures E3A, B**).

### EP4 Agonism Augments the Immunosuppressive Activity of MDSCs

**Figure 2:**
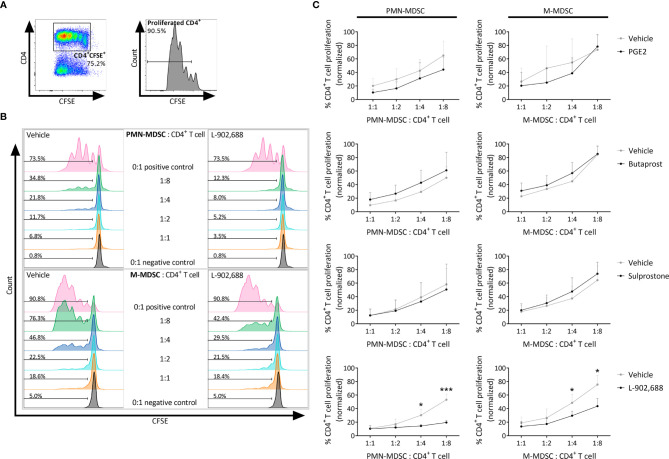
The effect of PGE2 and EP receptor agonists on the immunosuppressive activity of MDSCs. Bone marrow cells isolated from hind legs of 6- to 8-week-old female BALB/c mice were cultured in the presence of PGE2 or EP receptor agonists, including EP2 agonist Butaprost, dual EP1/EP3 agonist Sulprostone and EP4 agonist L-902,688 (all at 10 µM) or their respective vehicles, in combination with GM-CSF (20 ng/ml) and IL-6 (20 ng/ml). After 3 days of culture, both PMN- and M-MDSCs were isolated. CD4^+^ T cells were isolated from the spleens of 6- to 8-week-old female BALB/c mice. The MDSCs were then co-cultured with CFSE-labeled CD4^+^ T cells in the presence of anti-CD3/CD28. CFSE dilution was evaluated by flow cytometry after 3 days. **(A)** Gating strategy to assess CD4^+^ T-cell proliferation by flow cytometry. CFSE^+^CD4^+^ cells were first selected from viable cells after which the percentages of CD4^+^ T-cell proliferation were analysed (histogram). **(B)** Representative histograms showing the CFSE dilutions in CD4^+^ T cells that were co-cultured together with PMN- or M-MDSCs (pre-incubated with 10 µM L-902,688 or vehicle) at different ratios. **(C)** Analysis of the immunosuppressive activity of PMN- (left) and M-MDSCs (right). Data represents the normalised percentages of proliferating CD4^+^ T cells (positive control, without MDSCs, set to 100%). Data were pooled from 3-4 experiments with 2 technical replicates per experiment. Results are presented as mean ± SD. Statistical analysis was performed comparing each MDSC: CD4^+^ T cell ratio with unpaired two-tailed Student’s t-tests. *p < 0.05, ***p < 0.001 compared with vehicle.

To exclude the direct effect of PGE2 agonists on T cells, PGE2 and the EP receptor agonists were exclusively added during the preceding MDSC generation step and were absent from the MDSC - CD4^+^ T cell co-culture (**Figures 2A–C**). Strikingly, only PMN- and M-MDSCs that were generated in the presence of EP4 agonist L-902,688 significantly decreased CD4^+^ T-cell proliferation at a ratio of 1:8 compared to vehicle for PMN- and M-MDSCs, respectively. This augmented immunosuppressive effect was not observed in the MDSCs that were generated in the presence of other PGE2 agonists. As the EP4 agonist L-902,688 showed the most promising results, by enhancing the generation and augmenting the immunosuppressive activity of both PMN- and M-MDSCs, we continued our study by focusing on the EP4 receptor.

### EP4 Agonism Increases Arg1 and Nos2 Expression in MDSCs

**Figure 3:**
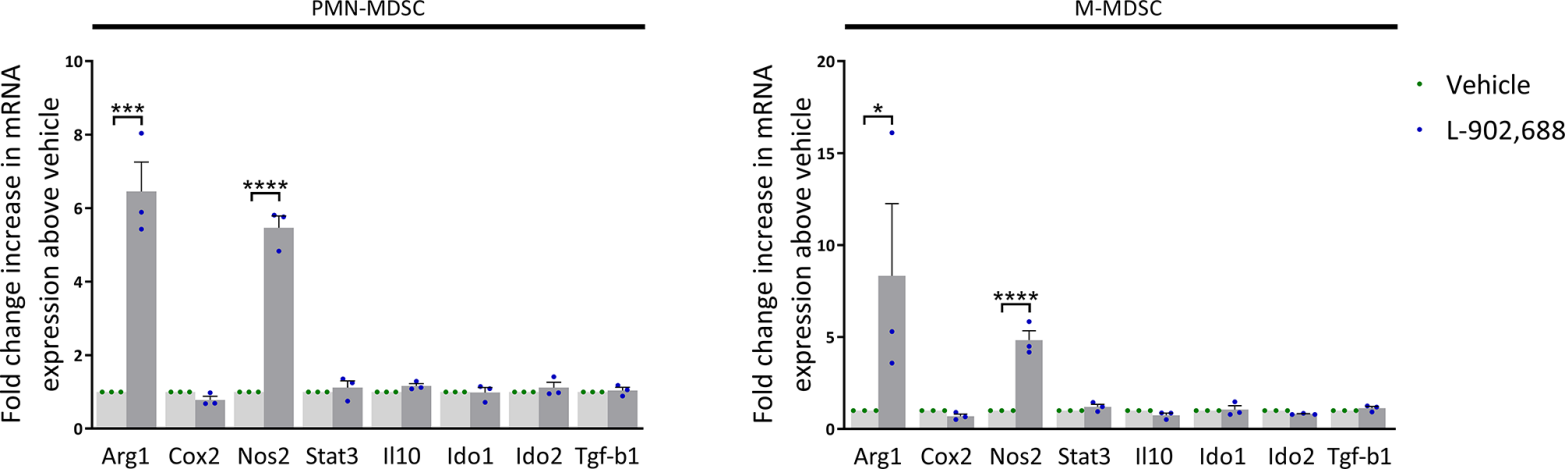
The effect of the EP4 agonist L-902,688 on mRNA expression of MDSCs. Bone marrow cells isolated from hind legs of 6- to 8-week-old female BALB/c mice were cultured in the presence of the EP4 agonist L-902,688 (10 µM) or vehicle, in combination with GM-CSF (20 ng/ml). After 3 days of culture, both PMN- and M-MDSCs were isolated. RNA was isolated and the gene expression of *Arg1*, *Cox2*, *Nos2*, *Stat3*, *Il-10*, *Ido1*, *Ido2* and *Tgf-b1* were quantified by qPCR. Expression shown as fold change increase in mRNA above vehicle. Data was normalised to *Gapdh* expression. Data was obtained from 3 independent experiments with 2 technical replicates per experiment. Results of individual experiments and mean ± SEM are shown. Statistical analysis was performed with unpaired one-tailed Student’s t-test. *p < 0.05, ***p < 0.001, ****p < 0.0001.

To study which pathways are activated during *in vitro* generation of MDSCs, cultured PMN- and M-MDSCs were isolated and further analyzed by qPCR. The gene expression of pathways of MDSC-mediated immune suppression were analyzed, including arginase (*Arg*) *1*, cyclooxygenase (*Cox*) *2*, nitric oxide synthase (*Nos*) *2*, signal transducer and activator of transcription (*Stat*) *3*, *Il-10*, indoleamine 2,3-dioxygenase (*Ido*) *1*, *Ido2* and transforming growth factor beta (*Tgf-b*) *1* (18). *Arg1* and *Nos2* expression was mainly upregulated in both PMN- and M-MDSCs exposed to the EP4 agonist L-902,688 (**Figure 3**). Due to the observed upregulation of *Arg1* expression in MDSCs after exposure to the EP4 agonist L-902,688, we hypothesized that MDSC/arginase-1 immune inhibition is one of the main pathways enhanced by the EP4 agonist.

### Adoptive Transfer of EP4-Primed MDSCs Ameliorates Pro-Inflammatory Cytokines Production in a Murine Model of Asthma

**Figure 4:**
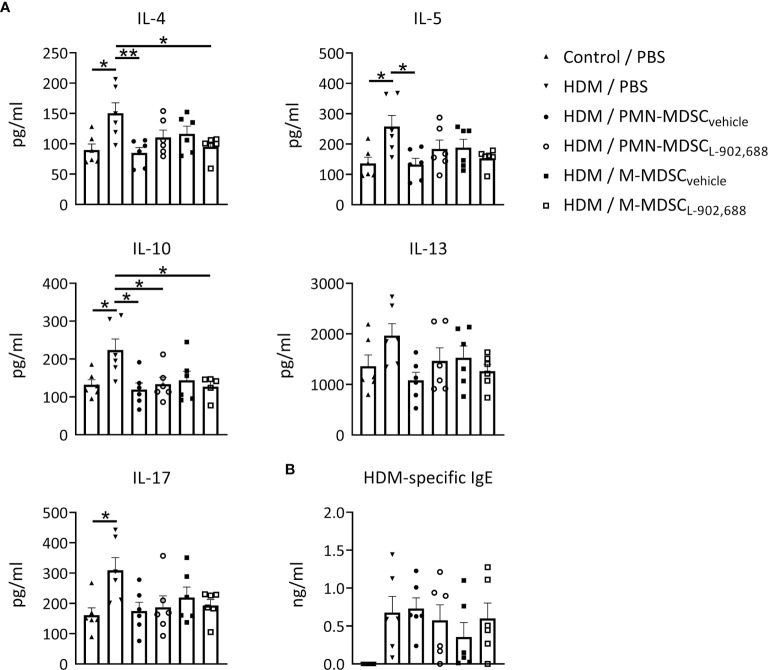
The effect of adoptively transferred MDSC_L-902,688_ on pro-inflammatory cytokine and IgE production in a murine model of asthma. 6- to 8-week-old female BALB/c mice were exposed to HDM or PBS on days 0, 7, 8, 9, 10 and 11. Bone marrow cells from hind legs of naïve female BALB/c donor mice were cultured in the presence of the EP4 agonist L-902,688 (10 µM) or vehicle in combination with GM-CSF (20 ng/ml) and IL-6 (20 ng/ml) for three days. Cultured PMN- and M-MDSCs were isolated and adoptively transferred (2x10^6^ of either PMN- or M-MDSCs) to the host mice on day 7 after HDM sensitization. Three days after the last HDM exposure **(A)** BAL supernatants were collected and examined for IL-4, IL-5, IL-10, IL-13, and IL-17A/F, and **(B)** serum supernatants were collected and examined for HDM-specific IgE levels using ELISAs. Data was obtained from 6 independent experiments with 2 technical replicates per experiment. Results of individual experiments and mean ± SEM are shown. Statistical analysis was performed with one-way ANOVA followed by Tukey’s multiple comparisons test. *p < 0.05, **p < 0.01.

We then assessed whether the adoptive transfer of PMN- and M-MDSC_L-902,688_, generated *in vitro* using the EP4 agonist L-902,688, would be able to alleviate lung inflammatory features in an HDM-induced murine model of asthma (**Figure E4A**). Pro-inflammatory cytokine production (IL-4, 5, 10, 13 and 17) in the BALF was reduced by adoptive transfer of both PMN- and M-MDSCs (**Figure 4A**). However, the adoptive transfer of PMN- and M-MDSC_L-902,688_ did not reduce these cytokine levels further compared to MDSC_vehicle_ (**Figure 4A**). HDM-specific IgE levels in the serum of asthmatic mice after adoptive transfer of MDSCs did not show any significant difference (**Figure 4B**). MDSC numbers in the lung, spleen, blood and bone marrow, 7 days after adoptive transfer, did not show significant differences in asthmatic mice (**Figures E4B, C**). The number of eosinophils in the BALF (**Figure E5**) and lung inflammatory features (**Figures E6A, B**) were reduced after adoptive transfer of MDSCs, but we again observed no significant differences in MDSC_L-902,688_ compared to MDSC_vehicle_.

### EP4 Agonism Enhances the Immunosuppressive Activity of Pulmonary MDSCs

**Figure 5:**
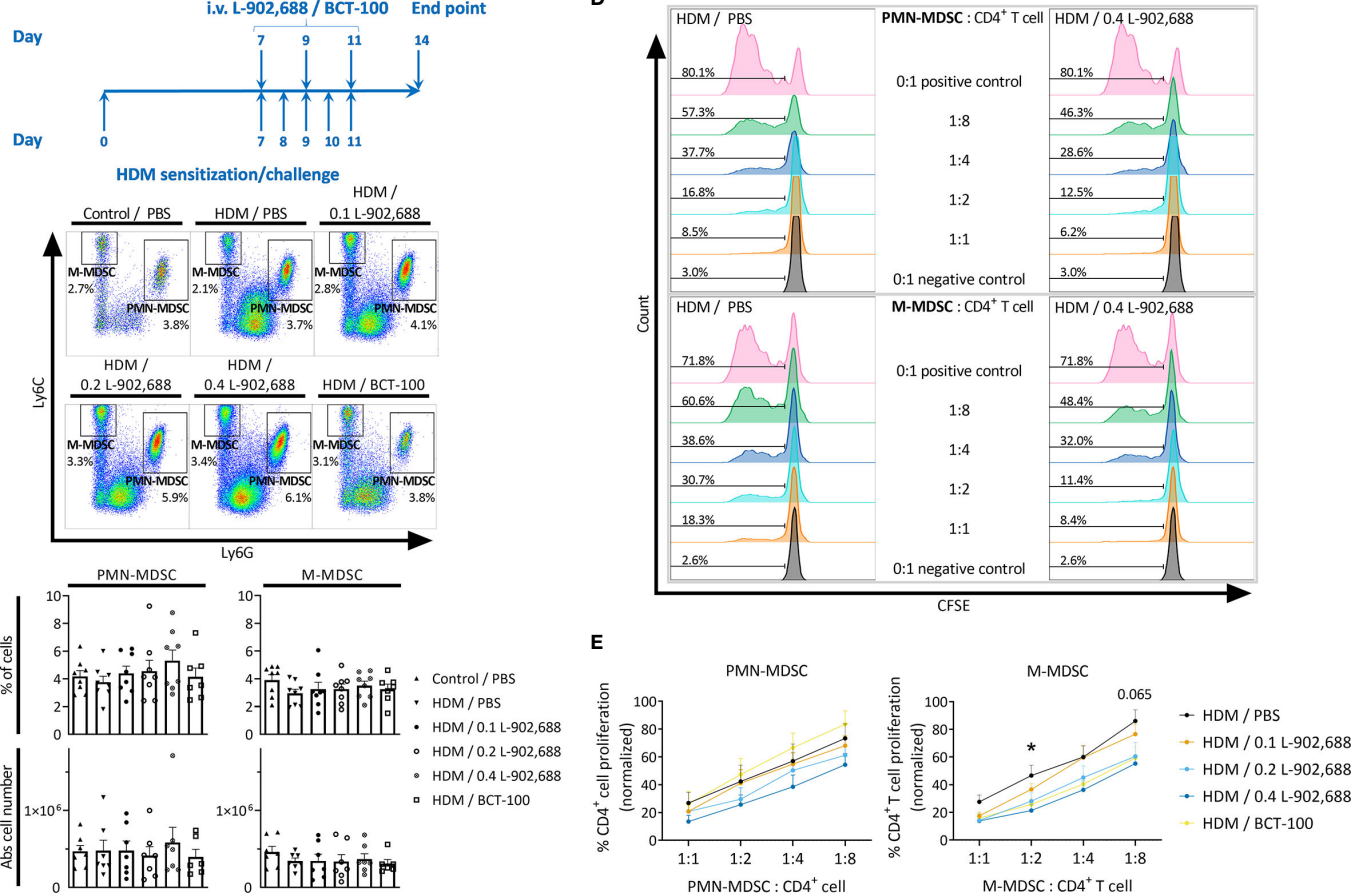
The effect of EP4 agonist L-902,688 and arginase-1 therapy on the number and activity of innate MDSCs in the lungs in a murine model of asthma. 6- to 8-week-old female BALB/c mice were exposed to HDM or PBS on days 0, 7, 8, 9, 10 and 11. On day 7, 9 and 11 of the HDM model, mice were intravenously administered the EP4 agonist L-902,688 (0.1; 0.2 or 0.4 mg/kg), BCT-100 (20 mg/kg) or PBS. Three days after the last HDM exposure the lungs were collected. **(A)** Representative timeline of the HDM-induced murine model of asthma and EP4 agonist L-902,688/BCT-100 therapy. **(B, C)** The number of PMN- and M-MDSCs in the lungs were assessed by flow cytometry. **(B)** Representative figures of MDSC analysis. **(C)** Data presented as the percentage and the absolute number of MDSCs in total lung cells. Data was obtained from 7-8 independent experiments with 1-2 technical replicates. Results of individual experiments and mean ± SEM are shown. **(D, E)** PMN- and M-MDSCs were isolated from the lungs and cultured together with CFSE-labeled CD4^+^ T cells isolated from the spleens of naïve donor mice in the presence of anti-CD3/28 for 3 days. CFSE dilution was assessed by flow cytometry. **(D)** Representative figures of the CFSE dilutions of gated CD4^+^CFSE^+^ cells at different ratios of PMN- or M-MDSC: CD4^+^ T cells. **(E)** Analysis of the immunosuppressive activity of MDSCs in the lung. Data represent the normalised percentages of proliferating CD4^+^ T cells (positive control, without MDSCs, set to 100%). Data were pooled from 5-6 independent experiments. Results are presented as mean ± SEM. Statistical analysis was performed, comparing each MDSC: CD4^+^ T cell ratio, with one-way ANOVA followed by Tukey’s multiple comparisons test. *p < 0.05 and *p* = 0.065, comparing EP4 0.4 mg/kg with HDM control.

Finally, we assessed the effect of three different doses of intravenously administered EP4 agonist L-902,688 in a murine model of asthma (**Figure 5A**). Furthermore, to examine the therapeutic effects of increased arginase-1 expression we decided to include a single dose of BCT-100, a pegylated recombinant arginase-1, as an *in vivo* therapy in a murine model of asthma (**Figure 5A**). We observed no significant change in the number of MDSCs in the lungs, except for the trend where the number of PMN-MDSCs increased with increasing doses of EP4 agonist L-902,688 (**Figures 5B, C**). Similar trends were observed in the number of PMN-MDSCs in the spleen, bone marrow and blood (**Figures E7A, B**). Both PMN- and M-MDSCs isolated from the lungs of asthmatic mice treated with the EP4 agonist L-902,688 showed a trend of increased immunosuppressive activity with increasing doses of agonist compared to MDSCs isolated from PBS-treated asthmatic mice (**Figures 5D, E**). M-MDSCs isolated from asthmatic mice treated with 0.4 mg/kg of EP4 agonist L-902,688 significantly reduced CD4^+^ T-cell proliferation by an average of 25% at a ratio of 1:2 (M-MDSC: CD4^+^ T cell) compared to PBS-treated asthmatic mice (**Figures 5D, E**). We observed a similar trend of increased immunosuppressive activity in M-MDSCs (not in PMN-MDSCs) isolated from asthmatic mice treated with BCT-100 (**Figures 5D, E**). The number of activated T cells (CD4^+^CD25^+^CD69^+^) in the lungs of asthmatic mice were reduced by treatment with 0.2 mg/kg of EP4 agonist L-902,688, although not significantly (**Figures E8A, B**). Pro-inflammatory cytokine production in the BALF showed slight trends of reduction after EP4 agonist L-902,688 and BCT-100 therapy (**Figure E9A**). HDM-specific IgE production in the serum of EP4 agonist L-902,688 and BCT-100 treated asthmatic mice are reduced, where asthmatic mice treated with 0.2 mg/kg EP4 agonist L-902,688 reached a significant reduction (**Figure E9B**).

### EP4 Agonism Ameliorates Lung Inflammation in a Murine Model of Asthma

**Figure 6:**
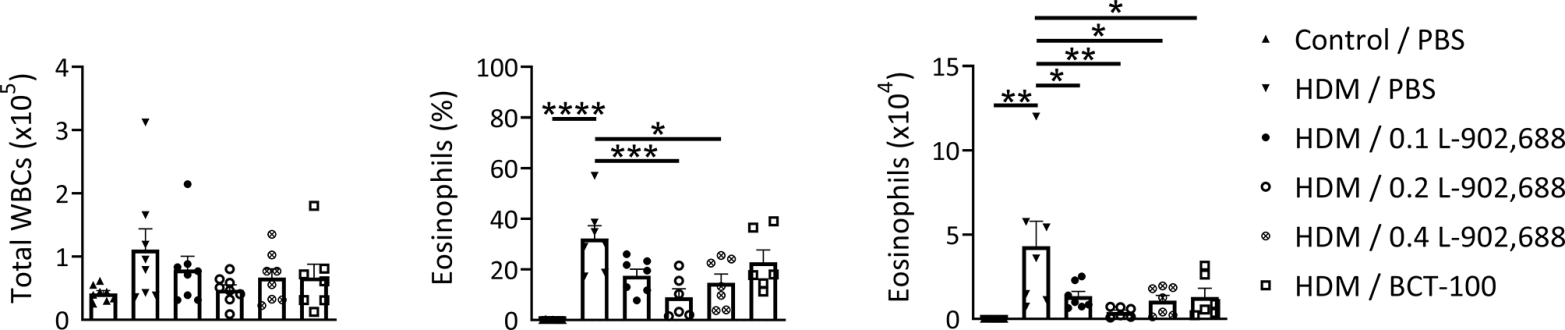
The effect of EP4 agonist L-902,688 and arginase-1 therapy on the number of total white blood cells (WBCs) and eosinophils in the BALF in a murine model of asthma. 6- to 8-week-old female BALB/c mice were exposed to HDM or PBS on days 0, 7, 8, 9, 10 and 11. On day 7, 9 and 11 mice were intravenously administered the EP4 agonist L-902,688 (0.1; 0.2 or 0.4 mg/kg), BCT-100 (20 mg/kg) or PBS. Three days after the last HDM exposure the BALF was collected. Differential cell counts were performed on cytospin preparations stained according to Pappenheim. Total number of WBCs, the percentage of eosinophils and the number of eosinophils are shown. Data was obtained from 6-8 independent experiments. Results of individual experiments and mean ± SEM are shown. Statistical analysis was performed with one-way ANOVA followed by Tukey’s multiple comparisons test. *p < 0.05, **p < 0.01, ***p < 0.001, ****p < 0.0001.

**Figure 7:**
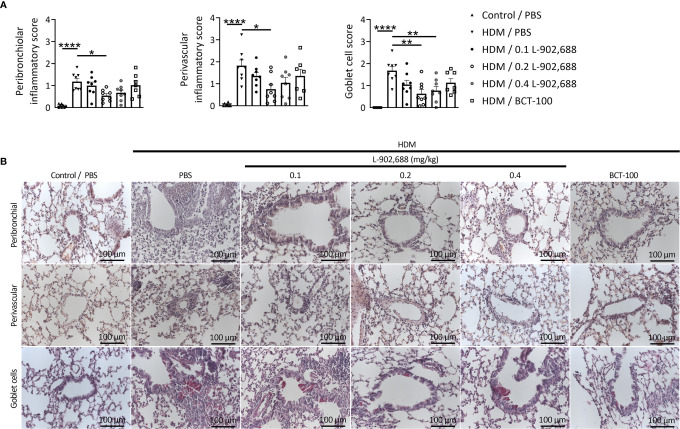
The effect of EP4 agonist L-902,688 and arginase-1 therapy on lung inflammatory features in a murine model of asthma. 6- to 8-week-old female BALB/c mice were exposed to HDM or PBS on days 0, 7, 8, 9, 10 and 11. On day 7, 9 and 11 of the HDM model, mice were intravenously administered the EP4 agonist L-902,688 (0.1; 0.2 or 0.4 mg/kg), BCT-100 (20 mg/kg) or PBS. Three days after the last HDM exposure the lungs were collected and used to assess lung inflammatory features. **(A, B)** Peribronchial and perivascular inflammatory scores were assessed by H&E staining and goblet cell score by PAS staining of lung sections. **(B)** Representative microscopic pictures are shown (40x magnification). Data was obtained from 7-8 independent experiments. Results of individual experiments and mean ± SEM are shown. Statistical analysis was performed with one-way ANOVA followed by Tukey’s multiple comparisons test. *p < 0.05, **p < 0.01, ****p < 0.0001.

The percentage and total number of eosinophils in the BALF were significantly reduced in asthmatic mice treated with the EP4 agonist L-902,688 compared to PBS-treated asthmatic mice (**Figure 6**). Total number of eosinophils in the BALF of asthmatic mice treated with BCT-100 significantly decreased as well. Lung inflammatory features were reduced in asthmatic mice treated with the EP4 agonist L-902,688 (**Figures 7A, B**). Peribronchial and perivascular inflammatory scores of the lungs of asthmatic mice treated with 0.2 mg/kg EP4 agonist L-902,688 were significantly reduced compared to PBS-treated asthmatic mice. Goblet cell scores were significantly reduced in the lungs of asthmatic mice treated with 0.2 and 0.4 mg/kg EP4 agonist L-902,688 compared to PBS-treated asthmatic mice.

## Discussion

MDSCs are important regulatory immune cells in a large variety of immune diseases, such as asthma (4, 5). We previously demonstrated that adoptive transfer of MDSCs through intratracheal and intravenous routes reduces airway inflammatory events in asthma models (4). Currently, mesenchymal stem cell (MSC) therapy is successfully used to treat patients with diseases such as graft *versus* host disease and liver cirrhosis (19). Furthermore, 68 clinical trials registered on the NIH Clinical Trial Registry website (clinicaltrials.gov) use MSC therapy to treat a range of pulmonary diseases, including asthma. In this regard, MDSC therapy could be a promising novel strategy considering the anti-inflammatory activity and lower risk of cell transformation in comparison to MSCs (20).

PGE2 has previously been used to enhance the generation of MDSCs from human PBMCs (21) and from murine bone marrow cells (22, 23) *in vitro*. Shi et al. showed that the effects of PGE2 on MDSC generation in murine bone marrow cells were likely mediated mainly through EP2 and, to a lesser extent, through EP4 receptor agonism (22). Consistent with these findings, we showed that PGE2 enhances the generation of GM-CSF/IL-6-induced PMN-MDSCs as well as M-MDSCs from murine bone marrow cells. However, in contrast to the work of Shi et al. (22) we found that this effect was mediated particularly through the EP4 receptor rather than the EP2 receptor. Adding to this finding, we could show the beneficial effects of EP4 agonism on allergic airway inflammation is not limited to PMN-MDSCs but applies to both PMN- and M-MDSCs.

Beyond MDSC generation, PGE2 has been found to play an important role in MDSC activation as well (24). Similarly, we found that PGE2 enhanced GM-CSF/IL-6-induced PMN- and M-MDSC activity through the EP4 receptor from cultured bone marrow cells. In line with these and related previous findings (23, 25–27), we found that PGE2 upregulated *Arg1* and *Nos2* expression in murine bone marrow cells through the EP2 and EP4 receptors. As previously discussed, arginase-1 is one of the main products of MDSCs that contribute to their anti-inflammatory effector functions. Increased arginase-1 and iNOS activity convert and deplete L-arginine deposits and thereby inhibit T-cell proliferation and T-cell function (28), while arginase-1 and nitric oxide (NO; product of iNOS) reduce MHC class II expression and NO induces cell apoptosis (29). However, the anti-inflammatory effect of EP4 agonism through arginase-1 needs to be confirmed using a selective arginase-1 inhibitor in an asthma model. Furthermore, ROS production, another immunosuppressive mechanism of MDSCs (30), needs to be studied in more detail, but was excluded from this study as ROS is not directly applicable as immunosuppressive therapy and therefore of lesser interest in this context.

Consistent with our previous work (7) and other studies (22, 31–33), we showed that the adoptive transfer of both MDSC_L-902,688_ and MDSC_vehicle_ ameliorated lung inflammatory features in a murine model of asthma. However, the increased immunosuppressive activity of MDSC_L-902,688_ over MDSC_vehicle_ shown in *in vitro* conditions did not translate to a significant reduction of lung inflammatory features *in vivo*. One can suggest that the time point of adoptive transfer and the number of MDSCs used for adoptive transfer should be further optimized. It is well known that the activity of MDSCs is largely dependent on the environment (5). For *in vitro* generated MDSCs, conditions are only controlled up until adoptive transfer. The immunosuppressive activity of *in vitro* generated MDSC_L-902,688_ might have been altered after adoptive transfer, due to complex *in vivo* conditions. The use of both the CD11b and Gr-1 (Ly6G and Ly6C) markers to characterize MDSCs in mice has previously been suggested and standardized by the recommendations of Bronte et al. (6). However, it is clear that MDSCs are a highly heterogenous cell population where the expression of certain markers largely depends on the type and state of disease, which together with the lack of specific markers further complicates the characterization of MDSCs (34). A recent study identified CD84 and Jaml as possible novel markers for MDSCs in breast cancer (15). In contrast to these findings we found that MDSCs cultured from murine bone marrow cells and in the presence of the EP4 agonist L-902,688, in fact do not express these markers (**Figure E10**). Nonetheless, based on the totality of previous work on MDSCs, we are confident that our analyses are comparable with previous lines of scientific evidence on murine MDSCs and can be interpreted accordingly.

Following our adoptive transfer study, we administered different doses of EP4 agonist to directly generate and activate intrinsic MDSCs. The bronchodilator and anti-inflammatory effects of PGE2 are known to be mediated through the EP4 receptor in humans (9, 35). However, the use of PGE2 in asthma is limited by various side effects, such as acute bronchoconstriction, soreness and cough, which are found to be primarily mediated by the EP1 and EP3 receptors (36–38). In this study, we show that EP4 agonist L-902,688 therapy increased immunosuppressive activity of intrinsic PMN- and M-MDSCs in a dose-dependent manner, correlating to our *in vitro* results. Reduced numbers of eosinophils observed in the BALF of asthmatic mice treated with the EP4 agonist L-902,688 are consistent with previous findings, where ONO-AE1-329, another selective EP4 agonist, inhibited eosinophil function and transendothelial migration (39, 40). In line with previous findings (22), we showed that the EP4 agonist L-902,688 ameliorates histological inflammatory features in the lung of asthmatic mice, by inhibiting inflammatory cell infiltration into both peribronchial and perivascular regions. The reduction of mucus-producing goblet cells is in line with previous findings where LPS-induced airway mucus hypersecretion was inhibited by EP4 receptor activation (41). The anti-inflammatory effects of EP4 therapy in the lungs of asthmatic mice, such as reduced eosinophil numbers and activated T cells as well as ameliorated airway histopathological changes, correlate to the effects that we and others observed after MDSC adoptive transfer (7, 22, 31, 32, 42, 43). To this end, these findings suggest that MDSCs play a key role in EP4 receptor-mediated anti-inflammatory effects in asthma. The EP4 receptor may therefore be a promising therapeutic target in asthma, in part, due to the anti-inflammatory effects mediated by MDSCs. Although, the bronchodilator effect of EP4 agonism is known in animals and in humans (9, 35), we were unable to measure lung function in our experimental setting and further clarify the translational potential in the context of asthma. Strikingly, until now, selective EP4 agonism has not been tested as a therapeutic agent in human asthma (44).

BCT-100 is a clinical-grade pegylated recombinant human arginase-1 with a plasma half-life of 3-4 days (45). Therapeutic effects and safety of BCT-100 were already shown in treating several types of cancer (45, 46). As mentioned above, we used BCT-100 to emulate anti-inflammatory effects of MDSCs in our asthma model. In line with EP4 agonist L-902,688 treatment, BCT-100 therapy showed a similar trend of airway anti-inflammatory effects in asthmatic mice. However, this finding needs to be studied in more detail, using different doses of BCT-100 and particularly in the presence of an arginase-1 inhibitor in asthma models.

In conclusion, here we provide evidence that MDSCs act anti-inflammatory in asthma *in vivo* through an EP4-mediated mechanism. In addition to its bronchodilator effect, EP4 receptor agonist-induced anti-inflammatory MDSCs may be a promising target to reduce inflammation in asthma. Furthermore, for the first time we showed that arginase-1 therapy may have beneficial effects in asthma. To this end, these findings may pave the way for EP4 receptor agonists, MDSC cell therapy and/or arginase-1 as potential new treatment approaches for asthma.

## Data Availability Statement

The raw data supporting the conclusions of this article will be made available by the authors, without undue reservation.

## Ethics Statement

The animal study was reviewed and approved by Regierungspräsidium Tübingen, Tübingen, Germany.

## Author Contributions

CG and SK designed the study, performed experiments, analyzed data, and wrote the manuscript. AD performed experiments. SB-H, BN, RH, HR, and DH provided scientific help and critically revised the manuscript. All authors contributed to the article and approved the submitted version.

## Funding

The project was funded by the *f*ortüne program of the University of Tübingen to S.K.; # 2458-0-0, # 2606-0-0. We acknowledge support by Open Access Publishing Fund of University of Tübingen.

## Conflict of Interest

DH was employed by Novartis.

The remaining authors declare that the research was conducted in the absence of any commercial or financial relationships that could be construed as a potential conflict of interest.
