# Supplementary material for: Myeloid-Derived Suppressor Cells Dampen Airway Inflammation Through Prostaglandin E2 Receptor 4
Source: Front Immunol. 2021 Jul 12;12:695933. doi: 10.3389/fimmu.2021.695933 (PMC8311661; doi:10.3389/fimmu.2021.695933)
Supplement: Supplementary file 1 [file DataSheet_1.docx]

Supplementary Material

# Supplementary Figures and Tables

## Supplementary Tables.

**TABLE E1|** **Primers used for qPCR analysis.**

| Genes |  | Primer sequence (5‘-3‘) |
| --- | --- | --- |
| *Gapdh* | Forward primer: | 5’- GCCTTCCGTGTTCCTACCC -3’ |
|  | Reverse primer: | 5’- CAGTGGGCCCTCAGATGC -3’ |
| *Arg1* | Forward primer: | 5'-ATTATCGGAGCGCCTTTCTC-3' |
|  | Reverse primer: | 5'-ACAGACCGTGGGTTCTTCAC-3' |
| *Cox2* | Forward primer: | 5’- CCTGGTGAACTACGACTGCT-3’ |
|  | Reverse primer: | 5’-GCCTGGGATGGCATCAGTT-3’ |
| *Nos2* | Forward primer: | 5'-CACCTTGGAAGAGGAGCAAC-3' |
|  | Reverse primer: | 5'-AAGGCCAAACACAGCATACC-3' |
| *Stat3* | Forward primer: | 5′-CCCGTACCTGAAGACCAAGT-3′ |
|  | Reverse primer: | 5′-ACACTCCGAGGTCAGATCCA-3′ |
| *Il-10* | Forward primer: | 5′-GAGAGCTGCAGGGCCCTTTGC-3′ |
|  | Reverse primer: | 5′-CTCCCTGGTTTCTCTTCCCAAGACC-3′ |
| *Tgf-b1* | Forward primer: | 5′-TGTACGGCAGTGGCTGAACCA-3′ |
|  | Reverse primer: | 5′-TGTCACAAGAGCAGTGAGCGCT-3′ |
| *Ido1* | Forward primer: | 5'-TCTGGGAATAAAACACGAGG-3' |
|  | Reverse primer: | 5'-GAAATGACAAACTCACGGAC-3' |
| *Ido2* | Forward primer: | 5'-CACAAGTACAACCACACAGA-3' |
|  | Reverse primer: | 5'-ATTTGGAAGGAGAAAGCCAT-3' |

## Supplementary Figure Legends.

**FIGURE E1| Purity of the isolated PMN- and M-MDSCs.** Bone marrow cells isolated from the hind legs of 6- to 8-week-old mice were treated with PGE2 agonists (10 µM) or their respective vehicles in the presence of GM-CSF (20 ng/ml) and IL-6 (20 ng/ml) for 3 days. PMN- or M-MDSC cells were isolated and analyzed by flow cytometry. CD11b^+^Ly6G^high^Ly6C^low^ and CD11b^+^Ly6G^-^Ly6C^high^ are defined as PMN-MDSCs and M-MDSCs, respectively. Data shown as the percentage of live cells. Data was obtained from 17 experiments with 1-2 technical replicates per experiment. Results from individual experiments and the mean ± SD are shown.

**FIGURE E2|** **The effect of PGE2 and EP receptor agonists on the immunosuppressive activity of MDSCs, including the direct effect on CD4^+^ T-cell proliferation.** Bone marrow cells isolated from the hind legs of 6- to 8-week-old female BALB/c mice were cultured in the presence of PGE2 or EP receptor agonists or their respective vehicles, in combination with GM-CSF (20 ng/ml) and IL-6 (20 ng/ml). After 3 days of culture, both PMN- and M-MDSCs were isolated. CD4^+^ T cells were isolated from the spleens of 6- to 8-week-old female BALB/c mice. The MDSCs were then co-cultured with carboxyfluorescein succinimidyl ester (CFSE)-labeled CD4^+^ T cells in the presence of the agonists and anti-biotin MACSiBead particles loaded with CD3ε- and CD28-biotin. CFSE dilution was evaluated by flow cytometry after 3 days. **A)** Gating strategy to assess CD4^+^ T-cell proliferation by flow cytometry. CFSE^+^CD4^+^ cells were first selected from viable cells before the percentages of CD4^+^ T-cell proliferation were analyzed (histogram). **B)** Representative plots showing the CFSE dilution in CD4^+^ T cells that were co-cultured together with PMN-MDSCs at different ratios. **C)** Analysis of the immunosuppressive activity of PMN-MDSCs (left) and M-MDSCs (right) in CD4^+^ T cell co-cultures, which were incubated with PGE2 or EP receptor agonists. The EP2 agonist Butaprost, dual EP1/EP3 agonist Sulprostone and EP4 agonist L-902,688 were used (1 µM, 10 µM). Data represents the normalized percentages of proliferating CD4^+^ T cells (positive control, without MDSCs, set to 100%). Data was pooled from 4-7 independent experiments with 1-2 technical replicates per experiments. Results are presented as mean ± SD. Statistical analysis was performed comparing each MDSC : CD4^+^ T cell ratio with repeated measures one-way ANOVA followed by Tukey’s multiple comparisons test. * p < 0.05, ** p < 0.01, *** p < 0.001, **** p < 0.0001 comparing 1 µM with vehicle; # p < 0.05, ## p < 0.01, ### p < 0.001, #### p < 0.0001 comparing 10 µM with vehicle; §§§ p < 0.001 comparing 1 µM with 10 µM.

**FIGURE E3|** **The direct effect of PGE2 and EP receptor agonists on CD4^+^ T-cell proliferation.** CD4^+^ T cells were isolated from the spleens of 6- to 8-week-old female BALB/c mice. Carboxyfluorescein succinimidyl ester (CFSE)-labeled CD4^+^ T cells were cultured in the presence of the agonists and anti-biotin MACSiBead particles loaded with CD3ε- and CD28-biotin. PGE2 (dissolved in DMSO), the EP2 agonist Butaprost (dissolved in methyl acetate), the dual EP1/EP3 agonist Sulprostone (dissolved in methyl acetate) and the EP4 agonist L-902,688 (dissolved in ethanol) were used at 1 µM and 10 µM. CFSE dilution was evaluated by flow cytometry after 3 days. Data represent the normalized percentages of proliferating CD4^+^ T cells (positive control, without MDSCs, set to 100%). Data was obtained from 4-5 independent experiments with 2-3 technical replicates per experiment. Results of individual experiments and mean ± SD are shown. Statistical analysis was performed with one-way ANOVA followed by Tukey’s multiple comparisons test. * p < 0.05, ** p < 0.01, *** p < 0.001, **** p < 0.0001 compared with respective vehicle, unless indicated otherwise.

**FIGURE E4|** **The effect of adoptively transferred MDSC_L-902,688_ on the number of innate MDSCs in a murine model of asthma.** 6- to 8-week-old female BALB/c mice were exposed to HDM or PBS on days 0, 7, 8, 9, 10 and 11. Bone marrow cells from hind legs of naïve female BALB/c donor mice were cultured in the presence of the EP4 agonist L-902,688 (10 µM) or vehicle in combination with GM-CSF (20 ng/ml) and IL-6 (20 ng/ml) for three days. Cultured PMN- and M-MDSCs were isolated and adoptively transferred to the host mice on day 7 after HDM sensitization. Three days after the last HDM exposure the lungs, spleen, bone marrow and blood were collected. **A)** Timeline of the acute HDM model of allergic airway inflammation and the MDSC_L-902,688_ adoptive transfer strategy. **B)** The number of PMN- and M-MDSCs in lung, spleen, blood, and bone marrow were assessed by flow cytometry. Data was obtained from 5-6 independent experiments with 1-2 replicates per experiment. **C)** The number of PMN- and M-MDSCs are presented as the percentage of total cells as well as the absolute cell number of the lung and spleen. Results of individual experiments and mean ± SEM are shown. Statistical analysis was performed with one-way ANOVA followed by Tukey’s multiple comparisons test. * p < 0.05.

**FIGURE E5| The effect of adoptively transferred MDSC_L-902,688_ on the number of total WBCs and eosinophils in the BALF in a murine model of asthma.** 6- to 8-week-old female BALB/c mice were exposed to HDM or PBS on days 0, 7, 8, 9, 10 and 11. Bone marrow cells from hind legs of naïve female BALB/c donor mice were cultured in the presence of the EP4 agonist L-902,688 (10 µM) or vehicle in combination with GM-CSF (20 ng/ml) and IL-6 (20 ng/ml) for three days. Cultured PMN- and M-MDSCs were isolated and adoptively transferred to the host mice on day 7 after HDM sensitization. Three days after the last HDM exposure the BALF was collected. Differential cell counts were performed on cytospin preparations stained according to Pappenheim. Total number of WBCs, the percentage of eosinophils and the total number of eosinophils are shown. Data was obtained from 5-6 independent experiments. Results of individual experiments and mean ± SEM are shown. Statistical analysis was performed with one-way ANOVA followed by Tukey’s multiple comparisons test. * p < 0.05, ** p < 0.01.

**FIGURE E6| The effect of adoptively transferred MDSC_L-902,688_ on lung inflammatory features in a murine model of asthma.** 6- to 8-week-old female BALB/c mice were exposed to HDM or PBS on days 0, 7, 8, 9, 10 and 11. Bone marrow cells from hind legs of naïve female BALB/c donor mice were cultured in the presence of the EP4 agonist L-902,688 (10 µM) or vehicle in combination with GM-CSF (20 ng/ml) and IL-6 (20 ng/ml) for three days. Cultured PMN- and M-MDSCs were isolated and adoptively transferred to the host mice on day 7 after HDM sensitization. Three days after the last HDM exposure the lungs were collected and used to assess lung inflammatory features. **(A, B)** Peribronchial and perivascular inflammatory scores were assessed by H&E staining and scoring the amount of inflammatory cells formed around the vessels and airways from grade 0 to grade 4 as follows: 0, normal lung; 1, scattered infiltration of a few inflammatory cells; 2, one ring of inflammatory cells; 3, two- to four layers of inflammatory cells; 4, more than four layers of inflammatory cells. PAS-positive mucus-containing goblet cells were semi-quantified by scoring from grade 0 to grade 4 as follows: 0, <5% PAS-positive cells; 1, 5-25%; 2, 25-50%; 3, 50-75%; 4, >75%. **B)** Representative microscopic pictures are shown. Data was obtained from 5-6 independent experiments. Results of individual experiments and mean ± SEM are shown. Statistical analysis was performed with one-way ANOVA followed by Tukey’s multiple comparisons test. * p < 0.05, ** p < 0.01.

**FIGURE E7| The effect of EP4 agonist L-902,688 and BCT-100 therapy on the number of innate MDSCs in the spleen, bone marrow and blood in a murine model of asthma.** 6- to 8-week-old female BALB/c mice were exposed to HDM or PBS on days 0, 7, 8, 9, 10 and 11. On day 7, 9 and 11 of the HDM model, mice were administered the EP4 agonist L-902,688 (0.1; 0.2 or 0.4 mg/kg, IV), pegylated recombinant human arginase-1 (BCT-100, 20 mg/kg, IV) or PBS. Three days after the last HDM exposure the lungs, spleen, bone marrow and blood were collected. The number of PMN- and M-MDSCs in the lungs, bone marrow and blood were assessed by flow cytometry and are presented as percentage of total cells as well as the absolute cell number in the spleen. Data was obtained from 7-8 independent experiments with 1-2 technical replicates per experiment. Results of individual experiments and mean ± SEM are shown.

**FIGURE E8| The effect of EP4 agonist L-902,688 and BCT-100 therapy on the number of active T cells in the lungs in a murine model of asthma.** 6- to 8-week-old female BALB/c mice were exposed to HDM or PBS on days 0, 7, 8, 9, 10 and 11. On day 7, 9 and 11 of the HDM model, mice were administered the EP4 agonist L-902,688 (0.1; 0.2 or 0.4 mg/kg, IV), pegylated recombinant human arginase-1 (BCT-100, 20 mg/kg, IV) or PBS. Three days after the last HDM exposure the lungs were collected. The number of active T cells in the lungs were assessed by flow cytometry. **A)** Representative figures of active T cell gating are shown, where CD4^+^ cells were gated prior to gating for CD25 and CD69. **B)** The number of active T cells (CD4^+^CD25^+^CD69^+^) as presented by percentage of total lung cells and absolute cell number. Data was obtained from 7-8 independent experiments with 1-2 technical replicates per experiment. Results of individual experiments and mean ± SEM are shown.

**FIGURE E9| The effect of EP4 agonist L-902,688 and BCT-100 therapy on pro-inflammatory cytokine and HDM-specific IgE production in a murine model of asthma.** 6- to 8-week-old female BALB/c mice were exposed to HDM or PBS on days 0, 7, 8, 9, 10 and 11. On day 7, 9 and 11 of the HDM model, mice were administered the EP4 agonist L-902,688 (0.1; 0.2 or 0.4 mg/kg, IV), pegylated recombinant human arginase-1 (BCT-100, 20 mg/kg, IV) or PBS. Three days after the last HDM exposure **(A)** the BAL supernatants were collected and examined for IL-4, IL-5, IL-13, and IL-17A/F, and **(B)** serum supernatants were collected and examined for HDM-specific IgE levels using ELISAs. Data was obtained from 5-8 independent experiments, with 2 technical replicates per experiment. Results of individual experiments and mean ± SEM are shown. Statistical analysis was performed with one-way ANOVA followed by Tukey’s multiple comparisons test. * p < 0.05.

**FIGURE E10| The expression of CD84 and Jaml in MDSCs generated from murine bone marrow cells in the presence of the EP4 agonist L-902,688.** Murine bone marrow cells isolated from the hind legs were cultured in the presence of the EP4 receptor agonist L-902,688 (10 µM) or vehicle, in combination with GM-CSF (20 ng/ml) and IL-6 (20ng/ml). After 3 days cells were subtyped for MDSCs and the expression of the CD84 and Jaml markers was analysed by FACS. Representative figures of gating are shown for both CD84 and Jaml in MDSCs, where CD11b^+^Ly6G^high^Ly6C^low^ and CD11b^+^Ly6G^-^Ly6C^high^ are defined as PMN- and M-MDSCs, respectively. Data is representative for the results of 3 experiments, with 2 technical replicates per experiment.
