# Supplementary material for: Myeloid-Derived Suppressor Cells Dampen Airway Inflammation Through Prostaglandin E2 Receptor 4
Source: Front Immunol. 2021 Jul 12;12:695933. doi: 10.3389/fimmu.2021.695933 (PMC8311661; doi:10.3389/fimmu.2021.695933)

FIGURE E1| Purity of isolated PMN- and M-MDSCs.

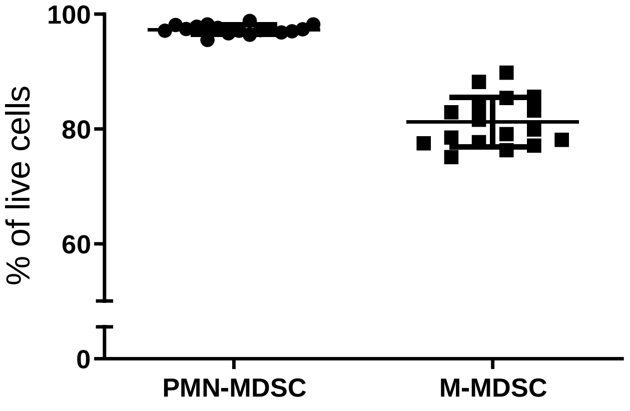

FIGURE E2| The effect of PGE2 and EP receptor agonists on the immunosuppressive activity of MDSCs, including the direct effect on CD4⁺ T cell proliferation.

A)

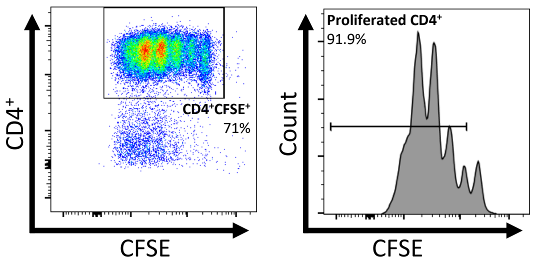

B)

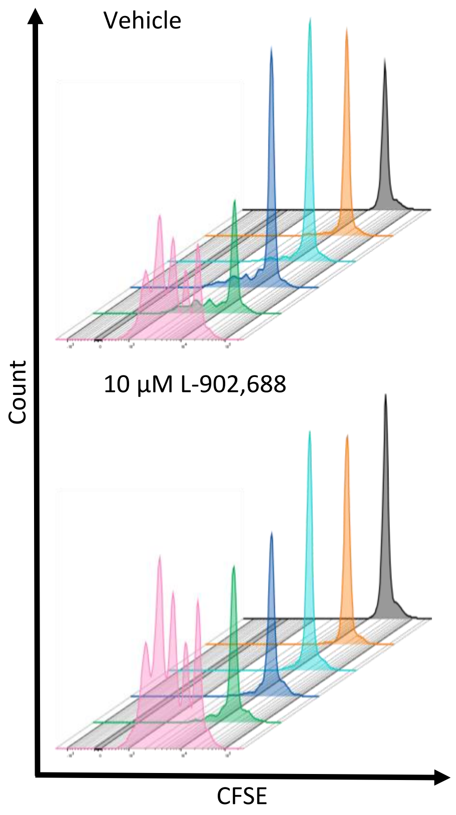

PMN-MDSC : CD4⁺ T cell	
0:1 negative control	4.8%
1:1	5.9%
1:2	8.8%
1:4	18.2%
1:8	44.7%
0:1 positive control	87.4%

PMN-MDSC : CD4⁺ T cell	
0:1 negative control	4.8%
1:1	6.9%
1:2	8.0%
1:4	8.7%
1:8	16.4%
0:1 positive control	87.4%

C)

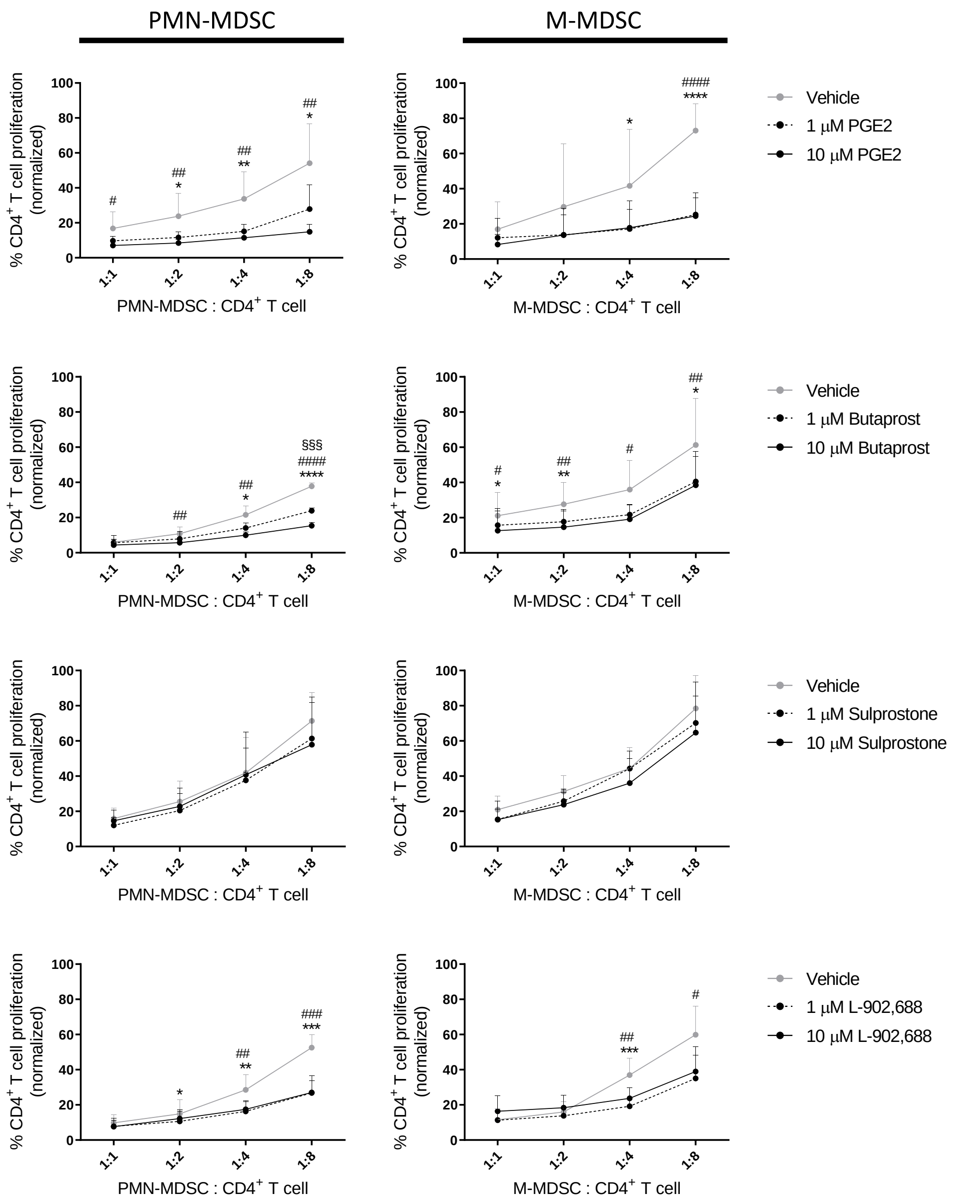

FIGURE E3| The direct effect of PGE2 and EP receptor agonists on CD4⁺ T cell proliferation.

A)

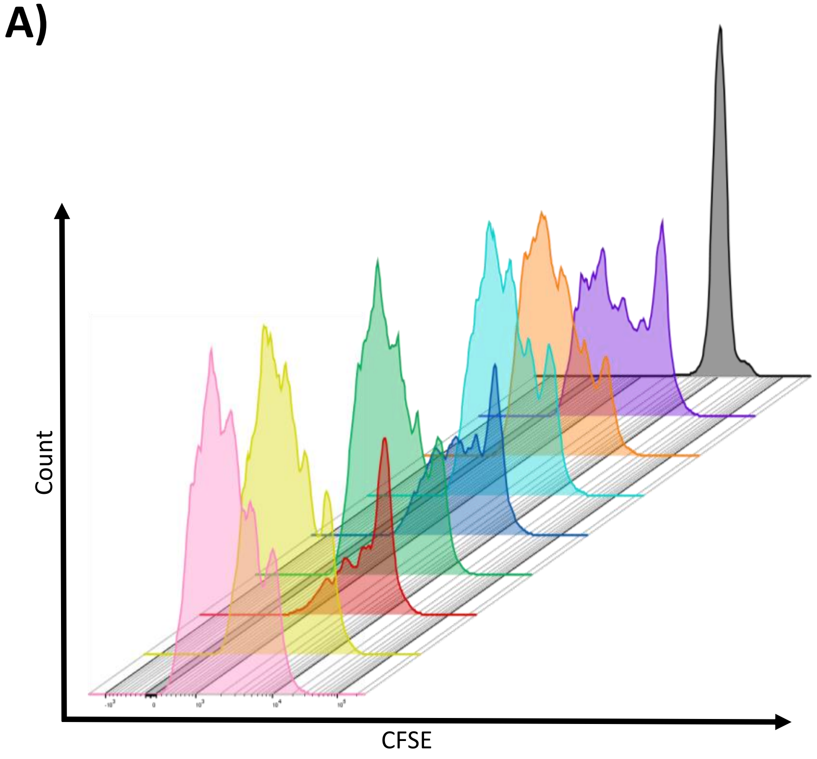

CFSE stained CD4⁺ T cells	
Negative control	2.9%
L-902,688 (10 µM)	72.2%
Ethanol (vehicle)	84.4%
Sulprostone (10 µM)	82.1%
Butaprost (10 µM)	63.4%
Methyl acetate (vehicle)	84.6%
PGE2 (10 µM)	43.6%
DMSO (vehicle)	84.0%
Positive control	84.5%

B)

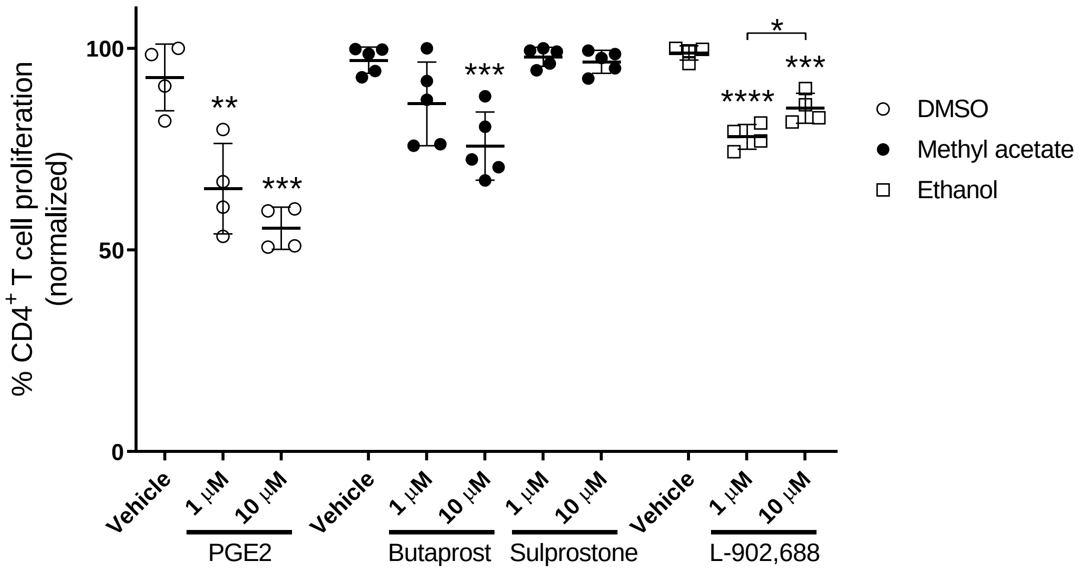

FIGURE E4| The effect of adoptively transferred $MDSC_{L\text{-}902,688}$ on the number of innate MDSCs in a murine model of asthma.

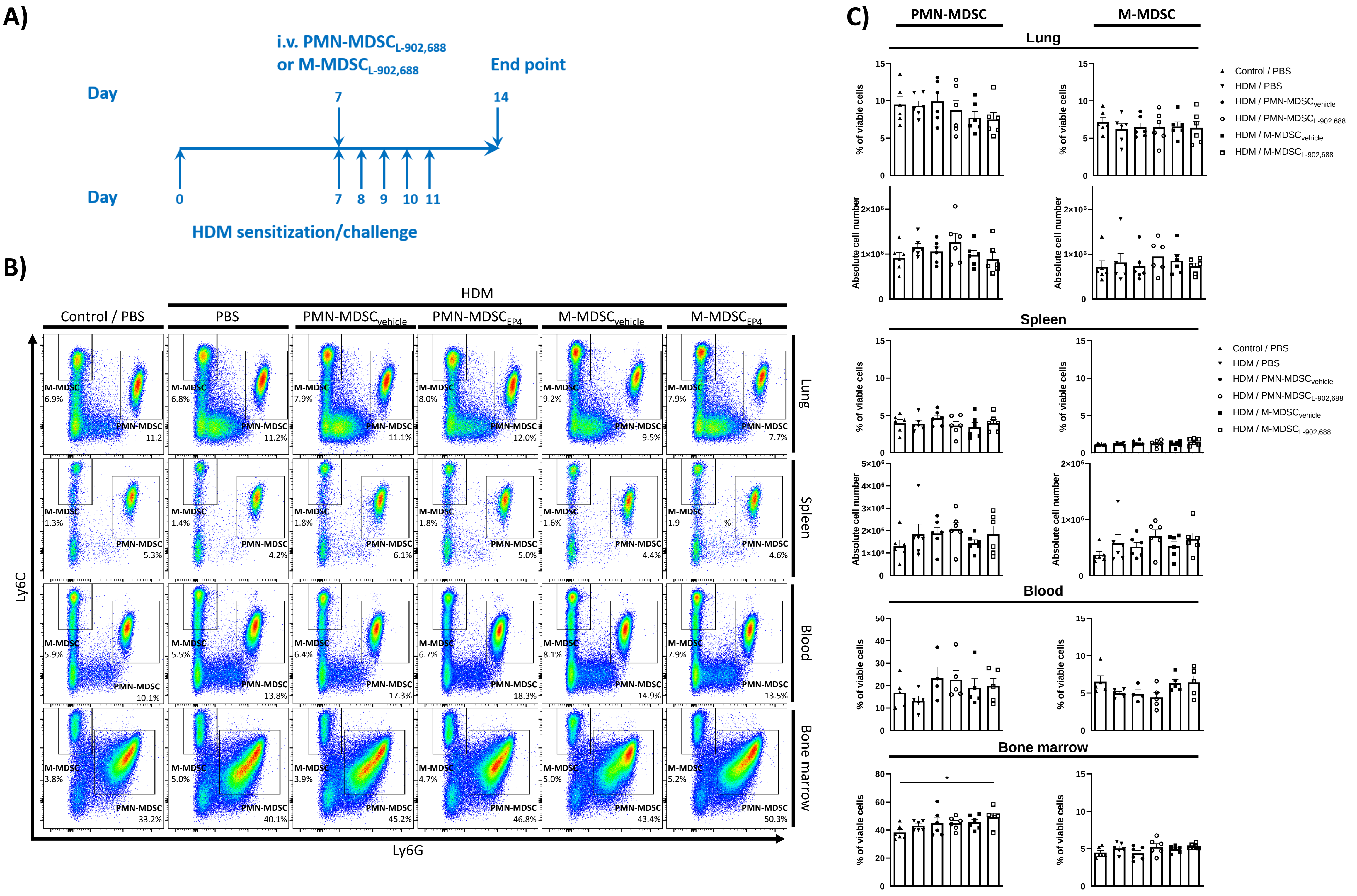

FIGURE E5| The effect of adoptively transferred MDSC$_{L-902,688}$ on the number of total WBCs and eosinophils in the BALF in a murine model of asthma.

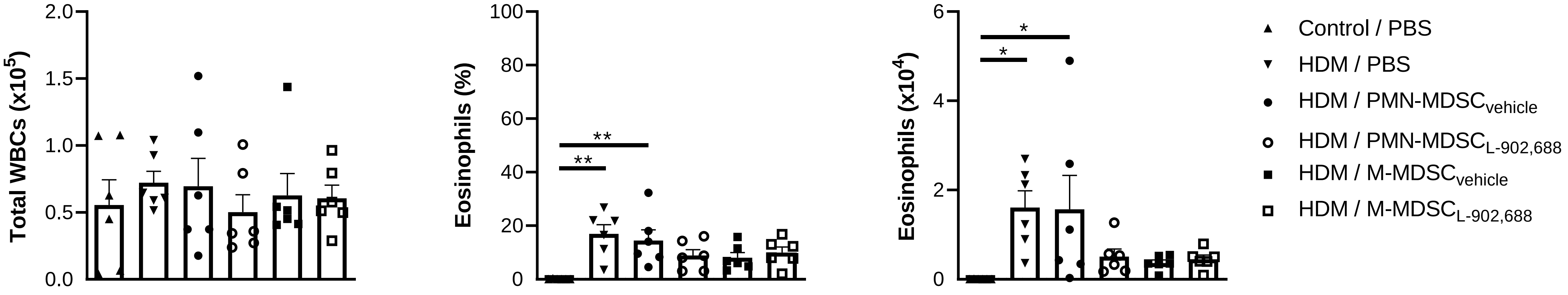

FIGURE E6| The effect of adoptively transferred $MDSC_{L-902,688}$ on lung inflammatory features in a murine model of asthma.

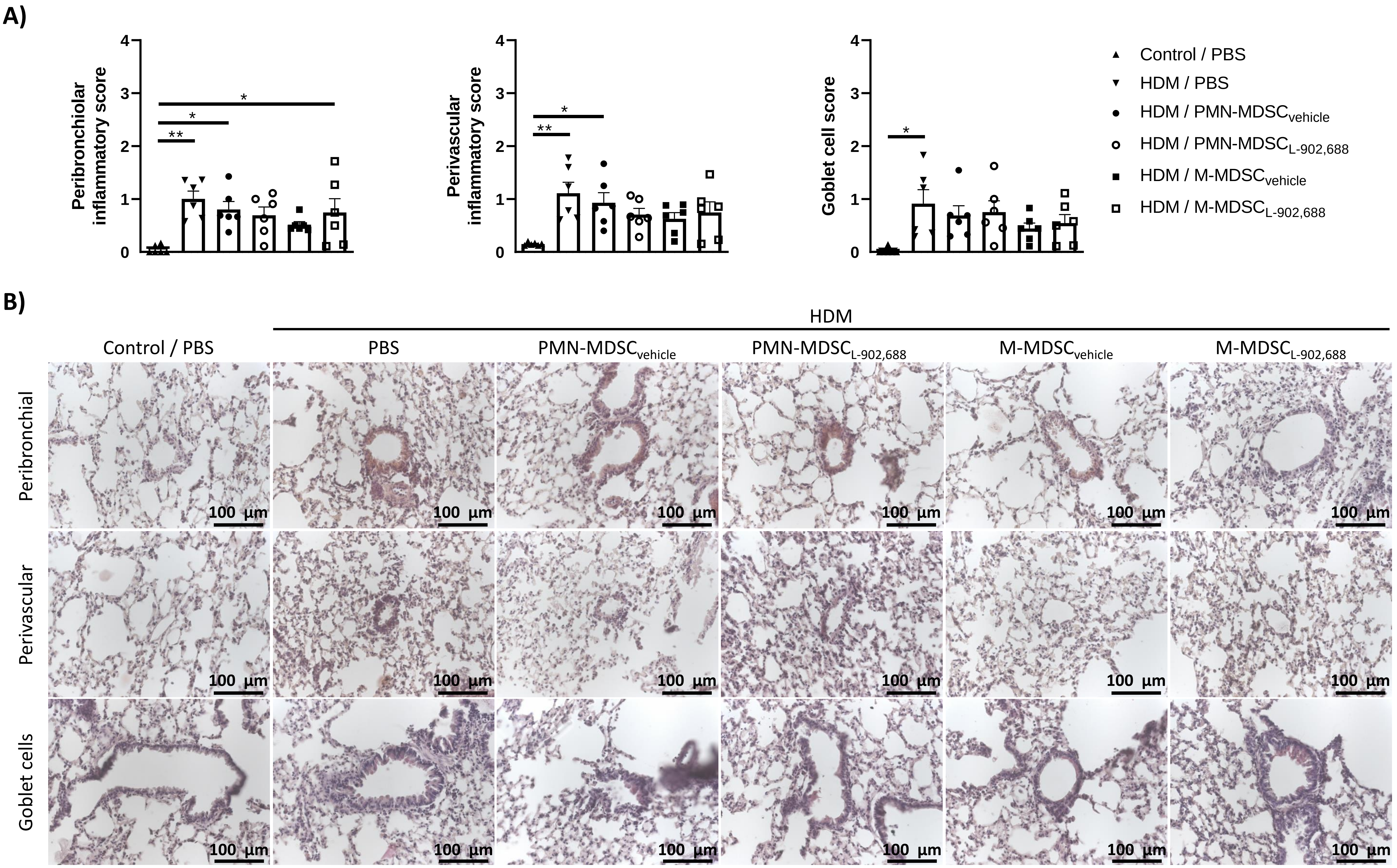

FIGURE E7| The effect of EP4 agonist L-902,688 and BCT-100 therapy on the number of innate MDSCs in the spleen, bone marrow and blood in a murine model of asthma.

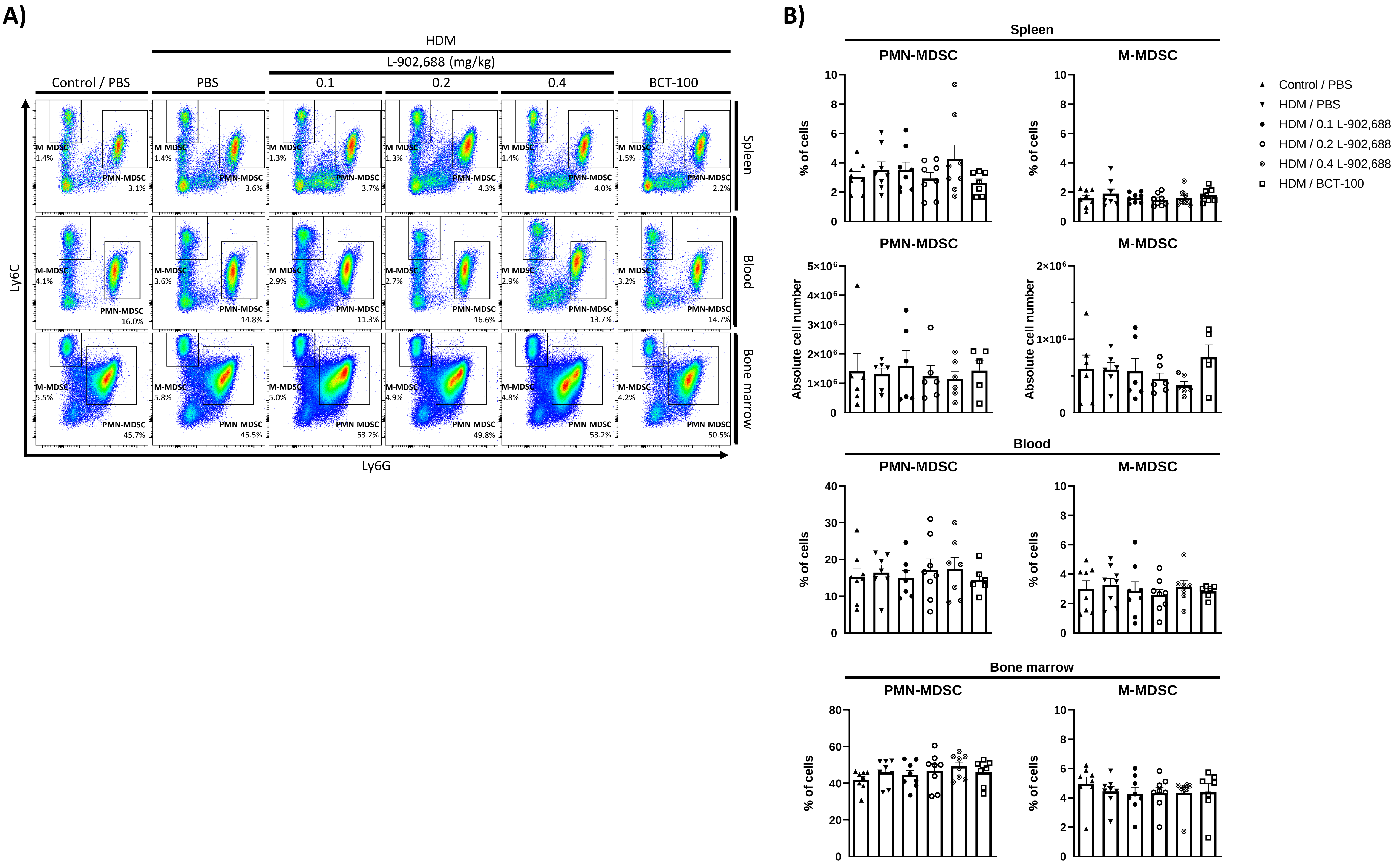

FIGURE E8| The effect of EP4 agonist L-902,688 and BCT-100 therapy on the number of active T cells in the lungs in a murine model of asthma.

A)

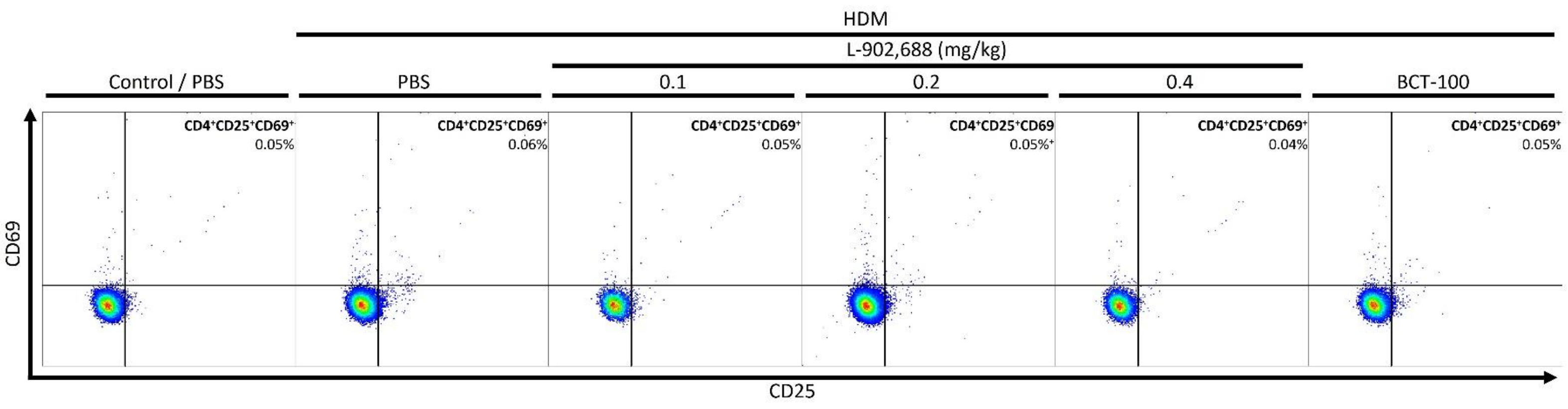

B)

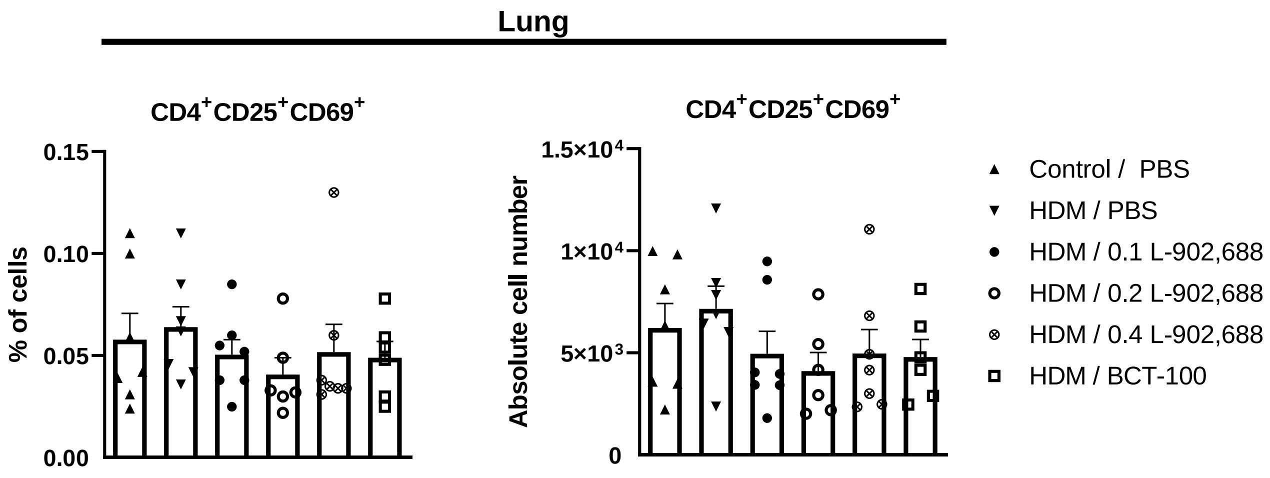

FIGURE E9| The effect of EP4 agonist L-902,688 and BCT-100 therapy on pro-inflammatory cytokine and HDM-specific IgE production in a murine model of asthma.

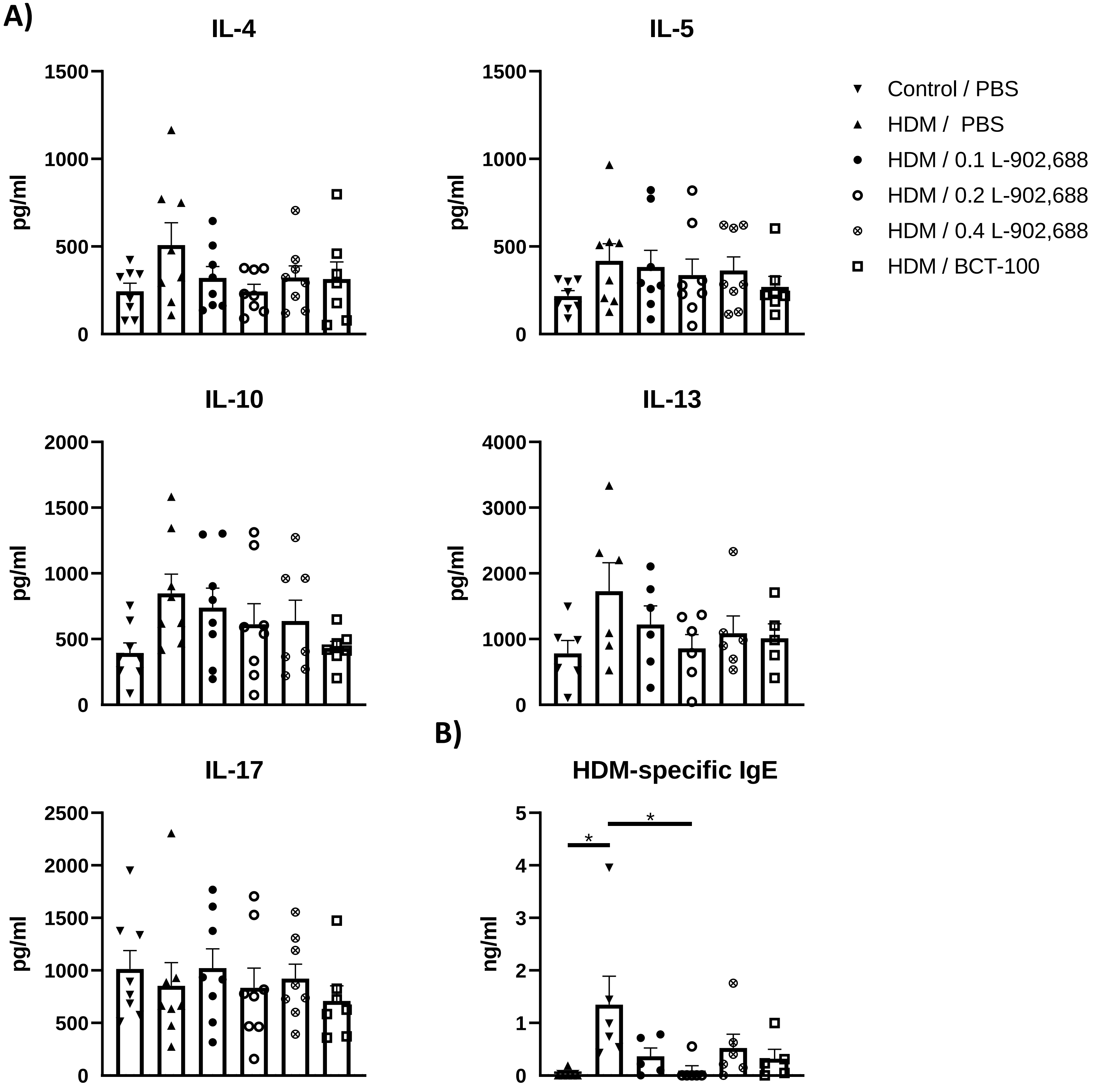

FIGURE E10| The expression of CD84 and Jaml in MDSCs generated from murine bone marrow cells in the presence of the EP4 agonist L-902,688.

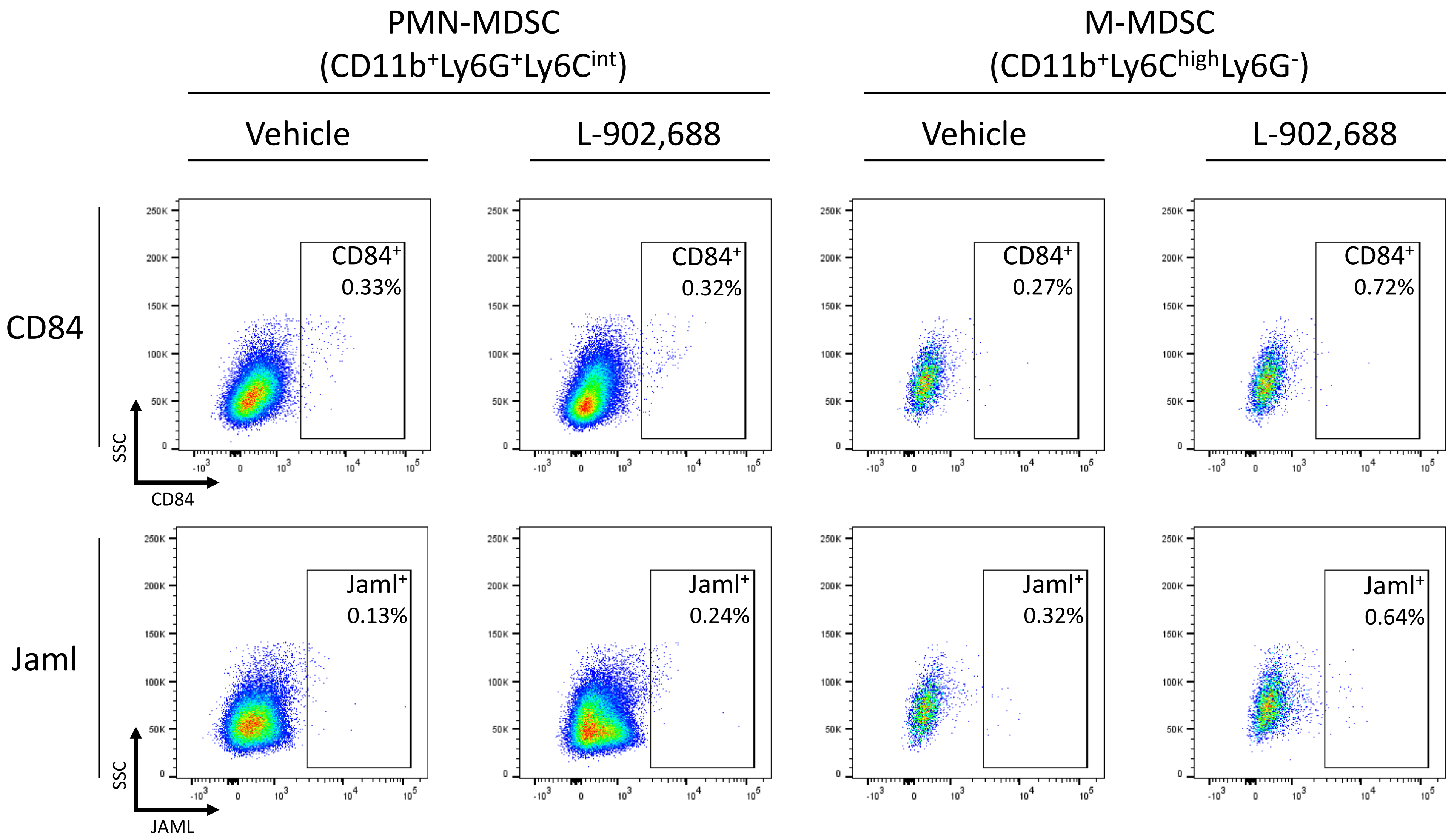

Supplement: Supplementary file 2 [file DataSheet_2.pdf]
